# A Very Low–Carbohydrate Program in Adults With Metabolic Dysfunction–Associated Steatotic Liver Disease and Phospholipase Domain–Containing Protein 3 Risk Genotype: Pre-Post Intervention Study

**DOI:** 10.2196/60051

**Published:** 2025-01-10

**Authors:** Laura R Saslow, Jamie Krinock, Alison O'Brien, Kaitlyn Raymond, Hovig Bayandorian, Judith T Moskowitz, Jennifer Daubenmier, Antonino Oliveri, Deanna J Marriott, Dina H Griauzde, Elizabeth K Speliotes

**Affiliations:** 1 Department of Health Behavior and Biological Sciences, School of Nursing, University of Michigan, Ann Arbor, MI, United States; 2 Department of Medical Social Sciences, Feinberg School of Medicine, Northwestern University, Chicago, IL, United States; 3 Institute of Holistic Health Studies, San Francisco State University, San Francisco, CA, United States; 4 Division of Gastroenterology, Department of Internal Medicine, University of Michigan Medical School, Medical Sciences Building II, Room 4741, Ann Arbor, MI, 48109, United States, 1 734-647-2964; 5 Department of Systems, Populations and Leadership, School of Nursing, University of Michigan, Ann Arbor, MI, United States; 6 VA Ann Arbor Healthcare System, Ann Arbor, MI, United States; 7 Department of Internal Medicine, University of Michigan Medical School, Ann Arbor, MI, United States; 8 Gilbert S Omenn Department of Computational Medicine and Bioinformatics, University of Michigan Medical School, Ann Arbor, MI, United States

**Keywords:** metabolic dysfunction–associated steatotic liver disease, ketogenic diet, low carbohydrate, adult, genotype, insulin, insulin resistance, metabolic dysfunction, dietary pattern, type 2 diabetes, T2DM, single-arm pilot trial, liver function test, genome, non-alcoholic fatty liver disease

## Abstract

**Background:**

Insulin resistance and the G allele of rs738409 interact to create a greater risk of metabolic dysfunction–associated steatotic liver disease.

**Objective:**

This study aims to confirm that one promising way to reduce insulin resistance is by following a very low–carbohydrate (VLC) dietary pattern.

**Methods:**

Adults with rs738409-GG or -CG with liver steatosis and elevated liver function tests, were taught an ad libitum VLC diet, positive affect and mindful eating skills, goal setting, and self-monitoring and given feedback and coaching for 4 months. We measured liver steatosis, anthropometric, serum metabolic diet adherence, and quality of life measures.

**Results:**

In this small pilot trial, of the 11 participants enrolled, 9 (82%) participants completed outcomes. All 11 participants viewed at least 1 session of the intervention, and 8 (73%) participants viewed at least half of the sessions. Among the 9 participants who provided 4-month self-report information, intervention satisfaction was high (mean 6.22, 95% CI 5.58-6.85), with 5 (56%) participants rating the intervention the top score, and 4 (44%) participants reporting they did not plan to stop following the VLC diet. Across participants with a 4-month hepatic liver fat percent measurement, the percent change in liver fat was −33.17% (95% CI −86.48 to 20.14), and in only the participants who were adherent to the eating pattern, the percent change in liver fat was −53.12% (95% CI −71.25 to −34.99). Amongst participants with a 4-month hepatic liver fat percent measurement, 6 out of 8 (75%) participants were considered responders, with a relative decline in liver fat ≥30%, and of the 9 participants with a 4-month body weight, 9 (100%) participants lost ≥5% of their body weight. There were no serious adverse events.

**Conclusions:**

Results suggest the feasibility, acceptability, and preliminary efficacy of the VLC intervention in adults with higher genetic risk for metabolic dysfunction–associated steatotic liver disease, although there is a need for further studies given the small sample size and the high risk of substantial biases in this small pilot study.

## Introduction

Metabolic dysfunction–associated steatotic liver disease (MASLD) is an increasingly prevalent disease afflicting about 25% of US adults and more than 50% of people with type 2 diabetes [1]. MASLD is caused by the deposition of excess fat in the liver, not due to alcohol, and can lead to advanced liver disease in the form of inflammation (metabolic dysfunction–associated steatohepatitis), fibrosis/cirrhosis (scarring), and hepatocellular carcinoma (liver cancer) [2]. Advanced MASLD is present in more than 90% of severely obese individuals [3]. It is associated with a shorter lifespan [4] and is expected to become the leading indication for liver transplantation in the United States [5].

Several dietary approaches have been suggested to treat MASLD, but no single approach has been found to be superior. For example, time-restricted eating and low-calorie, low-fructose, low-carbohydrate, very low–carbohydrate (VLC), and Mediterranean diets have all been studied in the context of MASLD [6-8].

We and others have shown that MASLD is heritable and the strongest common risk factor is having the I148M variant for the patatin-like phospholipase domain–containing protein 3 (PNPLA3) gene [9]. We have previously shown that individuals who carry this allele have a multiplicative risk of developing MASLD if they also have insulin resistance. The largest gene-environment meta-analysis study to date included approximately 15,000 individuals of diverse ancestry and demonstrated that rs738409-GG individuals had a 57% increased risk of MASLD if their insulin levels were in the highest versus lowest quartile of insulin, whereas rs738409-CC individuals had only a 32% increased risk, suggesting the presence of gene-environment interaction. Despite being at high risk of liver disease, individuals with rs738409-GG or -CG and insulin resistance are not being specifically targeted by programs aiming to reduce insulin resistance.

One promising way to reduce insulin resistance is through the use of a VLC eating pattern, also known as a ketogenic or “keto” eating pattern, which is a very reduced carbohydrate, moderate protein, and higher fat eating pattern. A VLC eating pattern has been found to be effective for reducing insulin resistance, body weight, inflammation, intrahepatic lipid content, and de novo fatty acid synthesis and fatty liver deposits, with some preliminary research showing the benefit of a VLC for MASLD [10-14]. On the other hand, high-carbohydrate, low-fat diets are related to greater liver inflammation or damage [15,16].

The National Institutes of Health’s obesity-related behavioral intervention trials model for behavioral intervention development [17] encourages preliminary testing of an intervention in a subpopulation before conducting a full-scale clinical trial. In this research, we built on previous research findings demonstrating that a VLC eating pattern can be taught using a web-based program that includes positive affect skills, which aim to increase the frequency with participants experience positive emotions, to improve intervention adherence and satisfaction [18,19]; mindful eating, to help participants cope with emotional and hedonic-driven eating, drivers of weight gain [20-22]; and other strategies including goal setting and self-monitoring; personalized feedback and support from a coach; social support; reminders; and booster messages [23].

We hypothesized that a VLC eating pattern might be able to achieve MASLD improvement or even reversal in adults with steatosis or mild fibrosis, especially in the higher-risk subpopulations of rs738409-GG or -CG individuals. As a first step, in this small pilot trial of adults with MASLD, we examined intervention feasibility, intervention acceptability, and physical and patient-reported outcomes of rs738409-GG or -CG individuals.

## Methods

### Study Design

This was a single-arm pilot trial of an online, VLC dietary intervention.

### Ethical Considerations

The research was approved by the University of Michigan Institutional Review Board (HUM00154361) and registered at ClinicalTrials.gov (NCT05010070). The study followed the ethical standards of the responsible committee on human experimentation (institutional and national) and abided by the Helsinki Declaration of 1975, as revised in 2000. All participants provided informed consent and could withdraw from the study at any point. No writing assistance was used for this manuscript. Recruitment began on August 31, 2021, and the outcomes of the trial were finalized by October 3, 2022.

### Participant Recruitment and Screening

Potential participants were contacted based on their rs738409 genotype in the Michigan Genomics Initiative (MGI), a research initiative that collects and genotypes blood samples and allows researchers to link this genetic information to patients. As part of the initiative, participants had already provided informed consent for broad research purposes [24]. Participants interested in our pilot study were referred to a webpage that described the trial and linked to an online screening survey (Qualtrics). The screening survey included questions used to assess eligibility. If individuals passed this, they were sent a video describing the goals, procedure, and pros and cons of participating in the trial. If they continued to express interest in the trial, they consented, baseline measures were collected, and then they were enrolled in the trial. Inclusion criteria included a baseline magnetic resonance imaging with liver steatosis but not cirrhosis, having been identified based on information from the MGI database, and having elevated liver function tests. Exclusion criteria included non-MASLD causes of elevated liver function tests; use of exogenous insulin; planned or history of weight loss surgery; active substance use or untreated mental health condition that could pose a safety risk; advanced medical conditions including as current chemotherapy, heart failure, or kidney failure; type 1 diabetes; Cushing syndrome; adherence to a vegan or dietary vegetarian; pregnant or planning to get pregnant in the next 6 months; currently enrolled in a weight loss program or other investigative study that might conflict with this research; taking medications known to cause weight gain or loss; or recent decompensation/hospitalization.

### Intervention

The online intervention included 16 weekly emails with a video and handouts, as well as text messages and email-based coaching. At baseline, participants received a body weight scale (Bodytrace) and urine ketone strips. Participants were also sent VLC cookbooks by mail 3 times throughout the intervention [25-27]. We taught participants to follow a VLC eating pattern, consisting of 20‐35 net (nonfiber) grams of carbohydrates per day. At week 6, we encouraged participants to become physically active for at least 150 minutes of moderate-intensity physical activity per week as well as to sleep enough to feel well rested. The intervention also taught skills related to feeling more positive affect and eating mindfully [28]. Throughout, we asked participants to track their dietary intake and to step on the body weight scale at least once a week. The program’s email-based coach provided feedback and support and we sent near daily, unique text messages that provided reminders of the intervention content.

### Data Collection

At baseline, participants were asked to self-weigh using the scale, complete a fasting blood draw and magnetic resonance imaging at Michigan Medicine, complete an online survey (Qualtrics); and complete a 2-day dietary recall with a trained dietitian by phone. At 4 months, at the end of the intervention, these were repeated. Prior to each week’s didactic session, we asked participants questions about their dietary adherence and experiences. Procedures associated with the research were either covered by the study or the participants’ insurance.

### Outcome Measures

#### Intervention Feasibility

##### Trial and Intervention Retention

We defined trial retention as the percentage of participants who completed the postintervention outcomes divided by the total number of trial participants enrolled. We assessed intervention retention based on intervention participation: (1) viewing at least 1 session, and (2) being active in the program, defined as viewing at least 8 out of 16 (50%) of the sessions.

##### Dietary Adherence

At baseline and 4 months, participants took part in two 24-hour dietary recalls. We considered participants adherent to the eating pattern if their net carbohydrates at 4 months were below 60 grams of carbohydrates per day and their overall calories did not increase by more than 400 kcal per day (as this was ad libitum, in which participants are asked to eat when hungry and stop when full, which typically leads to lower hunger).

### Intervention Acceptability

#### General Intervention Satisfaction

We asked participants, “How would you rate your overall satisfaction with the program?” Response options ranged from 1=“not at all satisfied” to 7=“very satisfied.” To assess potential acceptability, we also asked participants to answer, “How long can you see yourself following your assigned diet?”

#### Intervention Skills Satisfaction

Participants rated their satisfaction with the positive affect skills and, separately, the mindfulness skills. Response options ranged from 1=“don’t include them, they were not helpful” to 7=“you must include them, they were very helpful.”

#### Qualitative Feedback

At 4 months, using open-ended questions, we asked participants about their experiences with the intervention and recommendations for improvement.

### Patient-Centered Outcomes

#### Change in Liver Percent Fat and Fibrosis

We used magnetic resonance imaging to assess the percent fat of the left and right liver lobes, which we then combined. We also used magnetic resonance imaging to quantify the fibrosis in the liver. We examined what percent of participants lost at least 30% of their liver fat, a clinically significant threshold [29]. We a priori registered a reduction in liver fat as our primary outcome of the trial. We therefore examined this outcome for all participants and for adherent participants.

#### Body Weight Changes

Participants self-weighed throughout the trial and this weight was sent automatically to the trial’s staff through the scale’s connection to a cellular network. Mean change in weight and BMI (using baseline self-reported height) was calculated at 4 months compared with baseline. Mean percent weight loss was defined as (weight at 4 months – baseline weight) / (baseline weight) *100. We examined what percent of participants lost at least 5% of their body weight, a clinically significant threshold [30].

#### Blood Test Changes

We assessed liver function with aspartate aminotransferase and alanine aminotransferase, glucose control with glycated hemoglobin (HbA_1c_) and fasting glucose, fasting insulin, Homeostatic Model Assessment for Insulin Resistance (HOMA-IR), and lipids (triglycerides, high-density lipoprotein or HDL, and low-density lipoprotein or LDL), and inflammation (with C-reactive protein). We calculated the mean change at 4 months compared with baseline.

#### Self-Rated Change in Health

We asked participants to rate how their health changed over the intervention by answering the questions, “How much do you think your health has changed as a result of participating in this program?” (Response options ranged from 1=“very much worse” to 7=“very much better”).

#### Changes in Chronic Liver Disease Questionnaire (CLDQ)

We measured change in MASLD-related quality of life with the chronic liver disease questionnaire (CLDQ), which assesses 6 dimensions, rated from 1 to 7, with a higher number reflecting worse symptoms: abdominal symptoms (bloating, discomfort, and pain), activity (trouble with eating, physical ability), emotional (poor mood, sleep, and ability to concentrate), fatigue, systemic symptoms (body pain, dry mouth, itching, and muscle cramps), and worry about MASLD [31].

#### Changes in Psychological Factors

We assessed psychological changes from baseline to 4 months for positive affect with the Scale of Positive and Negative Experience [32], mindful eating with the reliance on hunger and satiety cues subscale of the Intuitive Eating Scale-2 [33], and stress-based eating with the Palatable Eating Motives Scale coping subscale [34].

### Statistical Analysis

For statistical significance, we followed the guidance for the use of frequentist inferential statistics in public health [35]. We completed an intention-to-treat analysis with all available data and analyses that were limited to only adherent individuals, with no imputation for missing data. Means and 95% CIs were calculated for continuous variables.

## Results

### Sample Characteristics

Of the 97 individuals who expressed interest in study participation and were screened for study eligibility, 55 did not meet inclusion criteria (including, as the top reasons for ineligibility: 13 were taking insulin, 11 had prior weight loss surgery or were taking weight loss medications, 8 had normal liver function tests, 6 had cirrhosis of the liver, 3 had heart failure or another related heart condition), 12 were eligible based on the prescreening survey but did not reply to follow-up requests, and 5 were eligible but declined to participate. Eleven participants enrolled in the trial.

At baseline, participants were 38‐77 years old (mean 55.54 years, SD 11.85 years), 8 out of 11 (72.7%) participants were male, most were White and not Hispanic (10/11, 90.9%), with 1 being White and Hispanic. Of the 11 participants, 7 (63.6%) participants were rs738409-GG and 4 (36.4%) participants were rs738409-CG. Other baseline values are in Table 1.

**Table 1.** Baseline for all participants (n=11).

Outcomes	Values
Age (years), mean (SD)	55.54 (11.85)
Sex (male), n (%)	8 (73)
Race (White and not Hispanic), n (%)	10 (91)
rs738409-GG (vs rs738409-CG), n (%)	7 (63.6)
Weight (kg), mean (95% CI)	96.19 (83.24-109.14)
BMI (kg/m^2^), mean (95% CI)	33.79 (29.60-37.98)
Liver lobe fat (%), mean (95% CI)	23.10 (14.25-37.98)
Liver lobe fibrosis, mean (95% CI)	2.46 (2.03-2.89)
AST^a^ (U/L), mean (95% CI)	49.91 (24.85-74.97)
ALT^b^ (U/L), mean (95% CI)	78.36 (36.90-119.83)
HbA_1c_^c^ (mmol/mol), mean (95% CI)	40.22 (35.86-44.58)
Glucose (mg/dL), mean (95% CI)	101.55 (91.44-111.65)
Insulin (mIU/mL), mean (95% CI)	21.15 (14.01-28.28)
HOMA-IR^d^, mean (95% CI)	5.44 (3.51-7.37)
Triglycerides (mg/dL) mean (95% CI)	127.82 (87.70-167.94)
HDL^e^ (mg/dL), mean (95% CI)	48.00 (35.56-60.44)
LDL^f^ (mg/dL), mean (95% CI)	84.82 (63.13-106.51)
C-reactive protein (mg/dL), mean (95% CI)	0.79 (0.24-1.34)
CLDQ^g^, mean (95% CI)	
Overall	2.62 (1.93-3.31)
Abdominal symptoms	2.49 (1.65-3.32)
Activity	2.33 (1.55-3.12)
Emotional function	2.60 (1.80-3.39)
Fatigue	2.98 (2.12-3.84)
Systemic symptoms	2.43 (1.67-3.19)
Worry about MASLD^h^	2.75 (1.78-3.73)
Positive affect, mean (95% CI)	24.18 (20.99-27.37)
Mindful eating, mean (95% CI)	2.88 (2.48-3.28)
Stress-based eating, mean (95% CI)	1.70 (1.24-2.17)
Energy, kilocalories, mean (95% CI)	1901.92 (1547.20-2256.64)
Carbohydrates (g), mean (95% CI)	192.40 (139.66-245.15)
Fiber (g), mean (95% CI)	17.23 (12.18-22.28)
Net (Non-fiber) Carbohydrates (g), mean (95% CI)	175.17 (123.97-226.37)
Total fat (g), mean (95% CI)	85.99 (67.55-104.43)
Monounsaturated fat (g), mean (95% CI)	30.38 (23.13-37.63)
Polyunsaturated fat (g), mean (95% CI)	17.26 (11.37-23.15)
Saturated Fat (g), mean (95% CI)	30.88 (23.74-38.02)
Protein (g), mean (95% CI)	80.31 (67.57-94.06)

a AST: aspartate aminotransferase.

b ALT: alanine aminotransferase.

c HbA_1c_: glycated hemoglobin.

d HOMA-IR: Homeostatic Model Assessment for Insulin Resistance.

e HDL: high-density lipoprotein.

f LDL: low-density lipoprotein.

g CLDQ: chronic liver disease questionnaire.

h MASLD: metabolic dysfunction–associated steatotic liver disease.

### Intervention Feasibility

#### Trial and Intervention Retention

Of the 11 participants who were enrolled, 9 (82%) participants completed study outcomes. All 11 participants viewed at least 1 session of the intervention, and 8 (73%) participants viewed at least half of the sessions.

#### Dietary Adherence

Nine participants completed the pre- and post-intervention dietary recalls. Changes in nutrient intake are shown in Table 2. In Table 3, we show results for just the 6 participants who were adherent to the eating pattern.

**Table 2.** Change in outcomes from baseline to post for all participants with outcome data. For all outcomes n=9, except for liver lobe fat % (n=8), liver lobe fibrosis (n=6), and C-reactive protein (n=4).

Outcomes	Baseline (mean, 95% CI)	Post (mean, 95% CI)	Percent change (mean, 95% CI)	Change (mean, 95% CI)
Weight (kg)	96.19 (83.24 to 109.14)	85.50 (74.63 to 96.37)	−10.88 (−13.06 to −8.69)	−10.69 (−13.37 to −8.00)
BMI (kg/m^2^)	32.93 (28.03 to 37.83)	29.26 (25.29 to 33.22)	−10.85 (−13.01 to −8.69)	−3.68 (−4.74 to −2.62)
Liver lobe fat (%)	19.13 (12.22 to 26.04)	12.06 (5.64 to 18.48)	−33.17 (−86.48 to 20.14)	−7.06 (−15.50 to 1.37)
Liver lobe fibrosis	2.41 (2.04 to 2.78)	2.09 (1.59 to 2.59)	−13.22 (−31.39 to 4.95)	−0.33 (−0.82 to 0.17)
AST^a^ (U/L)	50.22 (18.48 to 81.97)	41.33 (2.88 to 79.78)	−24.30 (−40.89 to −7.70)	−8.89 (−19.52 to 1.74)
ALT^b^ (U/L)	79 (26.35 to 131.65)	54.44 (−4.97 to 113.86)	−35.16 (−56.14 to −14.17)	−24.56 (−49.79 to 0.68)
HbA_1c_^c^ (mmol/mol)	40.22 (35.86 to 44.58)	36.33 (32.83 to 39.83)	−9.40 (−13.50 to −5.30)	−3.89 (−5.75 to -2.03)
Glucose (mg/dL)	105.56 (96.32 to 114.79)	95.56 (84.86 to 106.25)	−9.55 (−15.79 to −3.30)	−10.00 (−16.33 to -3.67)
Insulin (mIU/mL)	22.36 (14.01 to 30.7)	14.49 (9.04 to 19.94)	−30.71 (−55.17 to −6.25)	−7.87 (−15.44 to −0.29)
HOMA-IR^d^	5.88 (3.69 to 8.07)	3.54 (2.11 to 4.97)	−36.75 (−60.54 to −12.96)	−2.34 (−4.30 to −0.38)
Triglycerides (mg/dL)	123.89 (86.9 to 160.87)	107.89 (64.33 to 151.45)	−14.32 (−25.91 to −2.74)	−16.00 (−28.74 to −3.26)
HDL^e^ (mg/dL)	50.67 (35.63 to 65.7)	47.78 (37.57 to 57.99)	−2.50 (−14.87 to 9.88)	−2.89 (−9.62 to 3.84)
LDL^f^ (mg/dL)	80.11 (55.84 to 104.38)	83.44 (65.01 to 101.88)	9.33 (−9.12 to 27.78)	3.33 (−9.75 to 16.42)
C-reactive protein (mg/dL)	1.55 (0.59 to 2.52)	1.18 (0.36 to 2.00)	−23.58 (−59.94 to 12.77)	−0.38 (−1.07 to 0.32)
CLDQ^g^
Overall	2.33 (1.66 to 3.01)	1.68 (1.47 to 1.89)	−20.47 (−41.16 to 0.22)	−0.65 (−1.40 to 0.09)
Abdominal symptoms	2.67 (1.55 to 3.78)	1.37 (1.07 to 1.67)	−33.15 (65.25 to −1.05)	−1.30 (−2.48 to −0.11)
Activity	2.33 (1.49 to 3.17)	1.81 (1.16 to 2.47)	−9.88 (−45.53 to 25.77)	−0.52 (−1.58 to 0.55)
Emotional function	2.08 (1.58 to 2.58)	1.54 (1.21 to 1.87)	−22.55 (−39.55 to −5.55)	−0.54 (−1.11 to 0.03)
Fatigue	2.72 (1.62 to 3.83)	1.71 (1.13 to 2.29)	−27.05 (−49.50 to −4.59)	−1.01 (−2.02 to −0.01)
Systemic symptoms	2.31 (1.39 to 3.24)	1.82 (1.37 to 2.27)	−11.94 (−36.28 to 12.40)	−0.49 (−1.17 to 0.19)
Worry about MASLD^h^	2.18 (1.43 to 2.92)	1.82 (1.17 to 2.47)	−8.20 (−32.58 to 16.18)	−0.36 (−1.11 to 0.40)
Positive affect	25.56 (23.45 to 27.66)	26.78 (24.58 to 28.98)	5.01 (−1.11 to 11.13)	1.22 (−0.26 to 2.70)
Mindful eating	2.93 (2.44 to 3.41)	3.52 (3.24 to 3.79)	26.77 (−2.03 to 55.57)	0.59 (0.16 to 1.03)
Stress-based eating	1.69 (1.1 to 2.29)	1.31 (0.88 to 1.73)	−18.55 (−39.01 to 1.90)	−0.39 (−0.81 to 0.03)
Energy (kilocalories)	1752.25 (1401 to 2103.51)	1585.48 (1231.09 to 1939.88)	−6.97 (−29.49 to 15.55)	−166.77 (−551.21 to 217.67)
Carbohydrates (g)	171.37 (117.21 to 225.53)	88.53 (29.72 to 147.35)	−51.07 (−68.51 to −33.63)	−82.84 (−122.62 to −43.05)
Fiber (g)	17.35 (10.91 to 23.8)	18.88 (12.64 to 25.12)	22.28 (−28.19 to 72.76)	1.53 (−6.41 to 9.46)
Net (Non-fiber) Carbohydrates (g)	154.02 (102.54 to 205.5)	69.65 (13.66 to 125.65)	−58.62 (−76.36 to −40.89)	−84.36 (−123.63 to −45.09)
Total fat (g)	77.77 (60.71 to 94.83)	100.01 (71.62 to 128.4)	31.45 (−7.46 to 70.37)	22.24 (−4.99 to 49.47)
Monounsaturated fat (g)	28.2 (20.46 to 35.93)	34.43 (25.12 to 43.73)	25.24 (−4.70 to 55.18)	6.23 (−2.36 to 14.82)
Polyunsaturated fat (g)	13.95 (10.19 to 17.72)	18.47 (12.15 to 24.78)	41.43 (−22.53 to 105.39)	4.51 (−1.94 to 10.96)
Saturated fat (g)	28.53 (20.59 to 36.48)	38.48 (25.74 to 51.22)	38.58 (−5.55 to 82.71)	9.95 (−2.82 to 22.72)
Protein (g)	79.81 (63.11 to 96.51)	87.04 (72.31 to 101.76)	13.98 (−13.11 to 41.07)	7.22 (−13.07 to 27.51)

a AST: aspartate aminotransferase.

b ALT: alanine aminotransferase.

c HbA_1c_: glycated hemoglobin.

d HOMA-IR: Homeostatic Model Assessment for Insulin Resistance.

e HDL: high-density lipoprotein.

f LDL: low-density lipoprotein.

g CLDQ: chronic liver disease questionnaire.

h MASLD: metabolic dysfunction–associated steatotic liver disease.

**Table 3.** Change in outcomes from baseline to post for participants who were adherent to the eating pattern. For all outcomes n=6, except for C-reactive protein (n=3).

Outcomes	Baseline, mean (95% CI)	Post, mean (95% CI)	Percent change, mean (95% CI)	Change, mean (95% CI)
Weight (kg)	96.83 (83.93 to 109.74)	85.15 (74.36 to 95.94)	−11.99 (−14.33 to −9.65)	−11.69 (−14.59 to −8.78)
BMI (kg/m^2^)	34.05 (26.76 to 41.34)	29.92 (23.83 to 36.01)	−11.94 (−14.21 to −9.66)	−4.13 (−5.47 to −2.8)
Liver lobe fat (%)	21.67 (10.77 to 32.57)	10.92 (3.05 to 18.79)	−53.12 (−71.25 to −34.99)	−10.75 (−16.38 to −5.12)
Liver lobe fibrosis	2.35 (1.76 to 2.94)	1.88 (1.49 to 2.28)	−17.73 (−37.41 to 1.94)	−0.47 (−0.98 to 0.05)
AST^a^ (U/L)	41.67 (31.01 to 52.32)	26.33 (20.79 to 31.87)	−33.94 (−52.93 to −14.95)	−15.33 (−25.72 to −4.95)
ALT^b^ (U/L)	71 (35.6 to 106.4)	32.33 (22.52 to 42.15)	−47.49 (−70.67 to -24.31)	−38.67 (−70.38 to −6.95)
HbA_1c_^c^ (mmol/mol)	38.33 (34.48 to 42.18)	35.33 (31.6 to 39.07)	−7.73 (−13.15 to −2.32)	−3.00 (−5.10 to −0.90)
Glucose (mg/dL)	104.33 (90.18 to 118.49)	96.83 (80.54 to 113.12)	−7.36 (−15.76 to 1.05)	−7.50 (−15.65 to 0.65)
Insulin (mIU/mL)	25.3 (13.27 to 37.33)	15.08 (6.04 to 24.12)	−40.52 (−72.62 to −8.42)	−10.22 (−21.53 to 1.10)
HOMA-IR^d^	6.63 (3.43 to 9.82)	3.77 (1.45 to 6.09)	−44.02 (−77.02 to −11.03)	−2.86 (−5.79 to 0.08)
Triglycerides (mg/dL)	126.67 (64.35 to 188.98)	111.5 (38.45 to 184.55)	−13.59 (−32.80 to 5.61)	−15.17 (−36.73 to 6.40)
HDL^e^ (mg/dL)	50.83 (25.58 to 76.08)	47.33 (30.03 to 64.64)	−2.60 (−23.00 to 17.80)	−3.50 (−14.46 to 7.46)
LDL^f^ (mg/dL)	77 (50.1 to 103.9)	86.5 (63.52 to 109.48)	16.92 (−8.88 to 42.72)	9.50 (−5.70 to 24.70)
C-reactive protein (mg/dL)	1.83 (0.71 to 2.95)	1.43 (-0.57 to 3.44)	−17.16 (−74.65 to 40.33)	−0.40 (−1.71 to 0.91)
CLDQ^g^
Overall	2 (1.62 to 2.38)	1.7 (1.38 to 2.02)	−12.21 (−38.44 to 14.02)	−0.30 (−0.85 to 0.24)
Abdominal symptoms	2.44 (1.07 to 3.82)	1.28 (0.87 to 1.69)	−31.67 (−81.09 to 17.76)	−1.17 (−2.61 to 0.28)
Activity	1.89 (1.17 to 2.61)	1.67 (0.73 to 2.61)	−2.92 (−56.30 to 50.46)	−0.22 (−1.41 to 0.96)
Emotional function	1.88 (1.6 to 2.15)	1.65 (1.17 to 2.12)	−12.96 (−30.42 to 4.50)	−0.23 (−0.54 to 0.09)
Fatigue	2.12 (1.02 to 3.21)	1.57 (0.8 to 2.33)	−18.50 (−48.68 to 11.67)	−0.55 (−1.41 to 0.31)
Systemic symptoms	2 (1.36 to 2.64)	1.83 (1.26 to 2.4)	−3.96 (−39.18 to 31.27)	−0.17 (−0.75 to 0.42)
Worry about liver disease	1.93 (0.99 to 2.88)	2.03 (1.02 to 3.05)	5.42 (−7.76 to 18.59)	0.10 (−0.22 to 0.42)
Positive affect	25.83 (22.42 to 29.24)	27.33 (24.24 to 30.42)	6.28 (−3.59 to 16.15)	1.50 (−0.87 to 3.87)
Mindful eating	3.03 (2.62 to 3.43)	3.56 (3.1 to 4.01)	18.12 (5.48 to 30.75)	0.53 (0.15 to 0.90)
Stress-based eating	1.71 (0.75 to 2.67)	1.29 (0.66 to 1.92)	−20.79 (−39.39 to −2.20)	−0.42 (−0.85 to 0.01)
Energy (kilocalories)	1836.48 (1298.33 to 2374.62)	1385.79 (933.4 to 1838.17)	−24.28 (−38.40 to −10.16)	−450.69 (−777.83 to −123.54)
Carbohydrates (g)	169.25 (111.67 to 226.82)	60.25 (39.73 to 80.76)	−64.14 (−72.48 to −55.79)	−109.00 (−150.88 to -67.13)
Fiber (g)	19.11 (9.35 to 28.86)	16.86 (7.49 to 26.22)	−5.84 (−61.83 to 50.15)	−2.25 (−13.11 to 8.61)
Net (Non-fiber) Carbohydrates (g)	150.14 (95.45 to 204.83)	43.39 (28.69 to 58.08)	−70.04 (−79.77 to −60.31)	−106.75 (−153.97 to −59.54)
Total fat (g)	84.12 (58.22 to 110.01)	90.09 (49.99 to 130.19)	5.53 (−30.18 to 41.24)	5.97 (−23.95 to 35.90)
Monounsaturated fat (g)	30.1 (17.68 to 42.52)	32.04 (17.99 to 46.1)	7.89 (−29.08 to 44.86)	1.94 (−9.69 to 13.57)
Polyunsaturated fat (g)	15.64 (10.15 to 21.14)	14.96 (6.91 to 23)	−9.79 (−35.91 to 16.34)	−0.69 (−3.89 to 2.52)
Saturated fat (g)	30.58 (17.87 to 43.3)	35.17 (18 to 52.34)	17.23 (−32.59 to 67,06)	4.58 (−12.01 to 21.18)
Protein (g)	86.15 (60.68 to 111.63)	80.55 (61.78 to 99.33)	−4.24 (−23.72 to 15.24)	−5.60 (−25.7 to 14.51)

a AST: aspartate aminotransferase.

b ALT: alanine aminotransferase.

c HbA_1c_: glycated hemoglobin.

d HOMA-IR: Homeostatic Model Assessment for Insulin Resistance.

e HDL: high-density lipoprotein.

f LDL: low-density lipoprotein.

g CLDQ: chronic liver disease questionnaire.

### Intervention Acceptability

#### General Intervention Satisfaction

Of the 9 participants who provided 4-month self-report information, intervention satisfaction was high (mean 6.22, 95% CI 5.58-6.85), with 5 (56%) participants rating the intervention the top score. Only 1 (11%) participant reported that they would stop the assigned eating pattern as soon as the study was over, with 4 (44%) participants reporting that they intended to continue it for at least another few months, and 4 (44%) participants stating that they did not plan to ever stop following it.

#### Intervention’s Skills Satisfaction

The 9 participants who provided 4-month self-report information rated their satisfaction with the positive affect skills (mean 4.78, 95% CI 03.53-6.03), with 1 (11%) participant rating them the top score. Participants rated their satisfaction with the mindfulness skills (mean 4.44, 95% CI 3.09-5.79), with 1 (11%) participant rating them the top score.

#### Qualitative Feedback

Participants noted barriers that reduced their adherence to and motivation for the intervention, including lack of time, carbohydrate cravings, fatigue, vacations, and social situations. They mentioned factors that supported their adherence to and motivation for the intervention, including accountability by being in the intervention, the program’s supportive coach, feeling that the “diet was manageable,” weight loss success, and an overall desire to improve their own health. Opinions were mixed about the positive affect and mindful eating skills: one person said that they did not need them and another thought that the “skill building around mindfulness is essential.”

Participants noted positive health changes such as weight loss, increased energy, and a changed attitude about food and the “need/desire to eat food that is not good for me.” They noted that their family, friends, and physicians were impressed with their weight loss, health changes, and ability to stick with the program. Some comments included, “My physician noticed I lost weight and said I looked much better. She also encouraged me to continue this after the program is over.” “My primary care provider reported he was happy for the change and that my blood tests show I am now healthier than ever.”

### Secondary Patient-Centered Outcomes

#### Adverse Events

No serious adverse events were reported, but adverse events, possibly unrelated, included diarrhea (later discovered to be a Clostridioides difficile infection), a flare-up of dermatitis, and a broken foot.

#### Change in Liver Percent Fat, Body Weight, and Blood Tests

**Figure 1.**
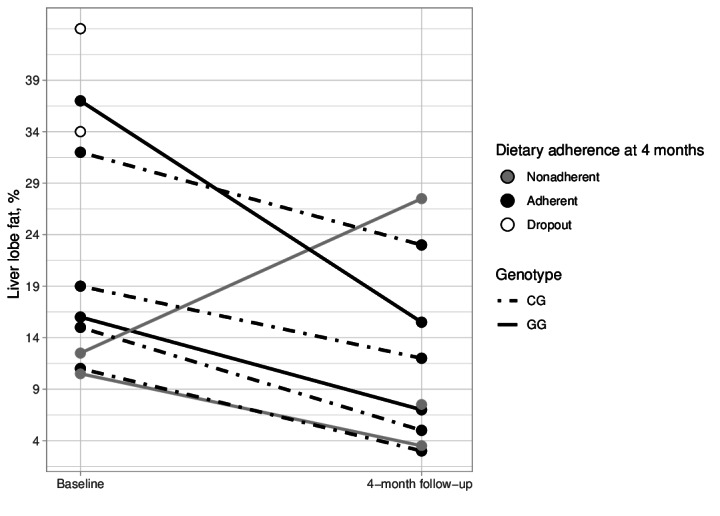
Change in liver lobe fat percent from baseline to post across participants.

In Figure 1 we show the changes in liver lobe fat percent across participants. In Table 2 we show the changes with values for all available participants. For the participants with this outcome, the percent change in liver fat was −33.17% (95% CI −86.48 to 20.14). In Table 3 we show these changes for only the participants who were adherent to the eating pattern. For the adherent participants, the percent change in liver fat was −53.12% (95% CI −71.25 to −34.99).

#### Achievement of ≥30% Liver Fat Loss

Amongst participants with a 4-month hepatic liver fat percent measurement, 6 out of 8 (75%) participants were considered responders, with a relative decline in liver fat ≥30%, with one person not counted losing 28%.

#### Achievement of ≥5% Body Weight Loss

Amongst participants with a 4-month body weight, 9 out of 9 (100%) of participants lost at least 5% of their body weight.

#### Self-Rated Change in Health

Two participants reported their health as having gotten a little better, 6 reported that their health was much better, and 1 reported that their health was very much better.

#### Changes in Chronic Liver Disease Questionnaire and Psychological Factors

Across all participants (Table 2) and for participants who were adherent (Table 3), changes were in the expected, salutary direction.

**Figure 2.**
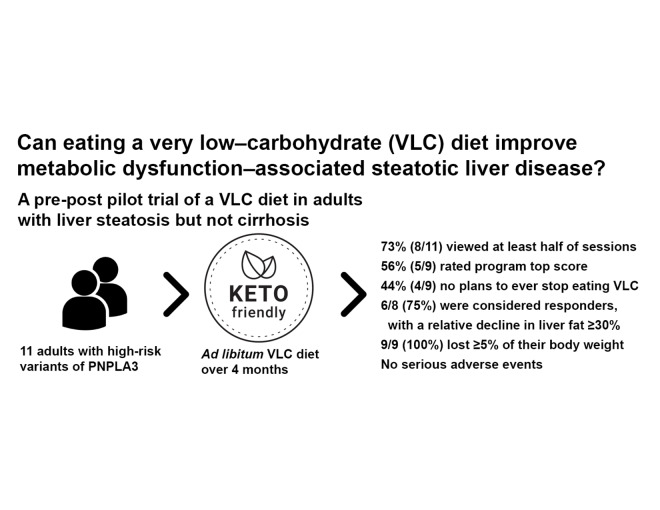
Graphical table of contents. PNPLA3: phospholipase domain–containing protein 3; VLC: very low–carbohydrate.

A summary of the study design and results is shown in Figure 2.

## Discussion

### Principal Findings

Results suggest the feasibility, acceptability, and preliminary efficacy of the VLC intervention in adults with higher genetic risk for MASLD, although there is a need for further studies given the small sample size and the high risk of substantial biases in this small pilot study. One concern of the VLC diet is its tolerability. Here we show that the diet was well tolerated with 5 out of 9 participants rating it at the highest satisfaction score. Only 1 out of 9 said they would stop the assigned dietary pattern as soon as the study was over. In contrast, 4 out of 9 said they would continue to follow the diet indefinitely. This is consistent with data where close to 60% of individuals with relapsing multiple sclerosis continued on a VLC or low-carb diet following a trial of the diet, particularly if they had lost a lot of weight and had decreased fatigue [36]. The mindfulness training was less helpful to participants with only 1 out of 9 rating it with the top score. They noted challenges with adhering to the diet as time, carbohydrate cravings, fatigue, vacations, and social situations which need to be addressed as part of a larger program to change eating patterns.

Several previous trials have examined the impact of a VLC eating pattern for adults with MASLD or metabolic dysfunction–associated steatohepatitis, and they found similar results. For example, in a 1-month trial of a VLC diet with 27 participants with MASLD, left hepatic lobe volume decreased by 20% [37]. In a 3-month trial of a Spanish and Mediterranean–adapted VLC diet in 12 men, liver steatosis improved in 93% of participants, and complete fatty liver regression was observed in 21% of participants [12]. In a 6-month trial of a VLC diet with 5 participants, 4 (80%) showed improvements in liver histology, steatosis, inflammatory grade, and fibrosis [10]. In a 2-month trial of a very low-calorie, VLC diet with 20 participants with obesity, the prevalence of liver steatosis (proton density fat fraction>5.4%) decreased from 70% to 30%. Likewise, 2 other nonrandomized, pre-post trials of hypocaloric VLC diets, a 6.5-month [38] and an 8-week trial, both showed improvements in liver steatosis [39].

Five previous trials, with a total of 179 people, have randomized with MASLD to a Mediterranean diet and measured liver-based fat with magnetic resonance imaging. These varied in length of time, with 1 being 1.5 months long, 2 being 3 months long, and 3 being 6 months long. Overall, they have found an average of 12%‐60% relative reduction in hepatic fat percentage [40-44]. Our results and the results of previous VLC trials suggest that a VLC, ketogenic diet may be at least as effective as a Mediterranean diet, although future randomized trials are needed to assess this. Our study is also in line with a previous study where carriers of PNPLA3 G risk allele were able to lose substantial liver fat on a low carbohydrate diet [45].

### Limitations

One of our major limitations is the fact that our sample size was small. Our recruitment was limited by the fact that we reached out to adults who had already been genotyped by the MGI. Instead, if we had genotyped interested participants, it may have been easier to recruit more participants. We also required that some of the study-related tests, which could be justified as medical expenses as part of regular health care, including the baseline magnetic resonance imaging and most of the blood tests, be covered by the participants’ health insurance. However, not all potential participants were willing to add these charges to their insurance, because it would still incur costs to the participants. In addition, the trial was conducted during the COVID-19 pandemic, which may have influenced who was willing or able to participate or their ability to adhere to dietary instructions.

Moreover, this trial’s generalizability is limited, as most participants were White, non-Hispanic men. Future recruitment efforts should focus on recruiting a more diverse population. This trial lacked a control group to which participants could have been randomized to, and thus statements cannot be made about causality.

Another important limitation is duration. As with other trials in this area, participants were tracked for less than a year, which does not allow for the understanding of the longer-term impacts of this nutritional approach nor whether participants may maintain dietary adherence longer term [10,12,37-44]. A growing body of literature including our paper showed that both insulin resistance and the PNPLA3 risk allele can drive the progression of liver disease [46,47]. A limitation of our study is that whether PNPLA3-driven liver fat accumulation causes liver insulin resistance cannot be determined here. Finally, the lack of a PNPLA3 CC control group limits our ability to determine whether the outcomes would be even more improved in the G versus C carriers.

### Conclusions and Future Directions

Thus, results supported the feasibility, acceptability, and preliminary efficacy of the VLC intervention in adults thought to have a higher genetic risk for MASLD. Overall, there is a need for longer-term studies to assess the sustainability of benefits and potential long-term risks associated with this way of eating. Moreover, future studies should aim to include a more diverse population, explore the biological mechanisms by which the VLC diet affects MASLD, consider the role of the PNPLA3 genotype, and integrate omics technologies to identify modulated biomarkers and pathways, thereby enhancing the generalizability and depth of the findings.

## Abbreviations

CLDQ: chronic liver disease questionnaire
MASLD: metabolic dysfunction–associated steatotic liver disease
MGI: Michigan Genomics Initiative
PNPLA3: phospholipase domain–containing protein 3
VLC: very low–carbohydrate
